# Crystal Structure of *Lymnaea stagnalis* AChBP Complexed with the Potent nAChR Antagonist DH*β*E Suggests a Unique Mode of Antagonism

**DOI:** 10.1371/journal.pone.0040757

**Published:** 2012-08-22

**Authors:** Azadeh Shahsavar, Jette S. Kastrup, Elsebet Ø. Nielsen, Jesper L. Kristensen, Michael Gajhede, Thomas Balle

**Affiliations:** 1 Department of Drug Design and Pharmacology, Faculty of Health and Medical Sciences, University of Copenhagen, Copenhagen, Denmark; 2 NeuroSearch A/S, Ballerup, Denmark; Virginia Commonwealth University, United States of America

## Abstract

Nicotinic acetylcholine receptors (nAChRs) are pentameric ligand-gated ion channels that belong to the Cys-loop receptor superfamily. These receptors are allosteric proteins that exist in different conformational states, including resting (closed), activated (open), and desensitized (closed) states. The acetylcholine binding protein (AChBP) is a structural homologue of the extracellular ligand-binding domain of nAChRs. In previous studies, the degree of the C-loop radial extension of AChBP has been assigned to different conformational states of nAChRs. It has been suggested that a closed C-loop is preferred for the active conformation of nAChRs in complex with agonists whereas an open C-loop reflects an antagonist-bound (closed) state. In this work, we have determined the crystal structure of AChBP from the water snail *Lymnaea stagnalis* (*Ls*) in complex with dihydro-*β*-erythroidine (DH*β*E), which is a potent competitive antagonist of nAChRs. The structure reveals that binding of DH*β*E to AChBP imposes closure of the C-loop as agonists, but also a shift perpendicular to previously observed C-loop movements. These observations suggest that DH*β*E may antagonize the receptor via a different mechanism compared to prototypical antagonists and toxins.

## Introduction

Neuronal nicotinic acetylcholine receptors (nAChRs) are pentameric ligand-gated ion channels present both in the central and peripheral nervous system. nAChRs belong to the Cys-loop superfamily and exist as homo or heteromeric receptors composed of either *α* subunits or *α* and *β* subunits in combination. The subunits are arranged symmetrically around a central ion channel pore. Each monomer possesses an *N*-terminal extracellular ligand-binding domain, a transmembrane region that forms the ion channel pore, and an extended intracellular loop [1]–[3]. These receptors are allosteric proteins that exist in a minimum of three different conformational states, termed the resting (closed), activated (open) and desensitized (closed) states. The balance between these states regulates the permeability of cations through the ion channel [4].

Insight into ligand binding and nAChR activation is rapidly emerging and structures of the acetylcholine binding protein (AChBP) have significantly aided this process. The mulloskan AChBP is a structural and functional homologue of the extracellular domain of nAChRs [5], [6]. Previous studies have suggested that a closed C-loop is associated with agonist-bound structures of AChBP, and thus represents an active conformation of nAChRs, whereas an open conformation of the C-loop observed in antagonist-bound structures represents an inactive form of the receptor [7]–[10]. Likewise, a correlation between the degree of agonism and closure of the C-loop has been suggested [8], [11]. In contrast to this, it was reported in a recent study that a series of agonists with 21–76% efficacy at *α*4*β*2 nAChRs displayed no variation in the degree of C-loop closure in *Lymnaea stagnalis* (*Ls*) AChBP [12].

**Figure 1:**
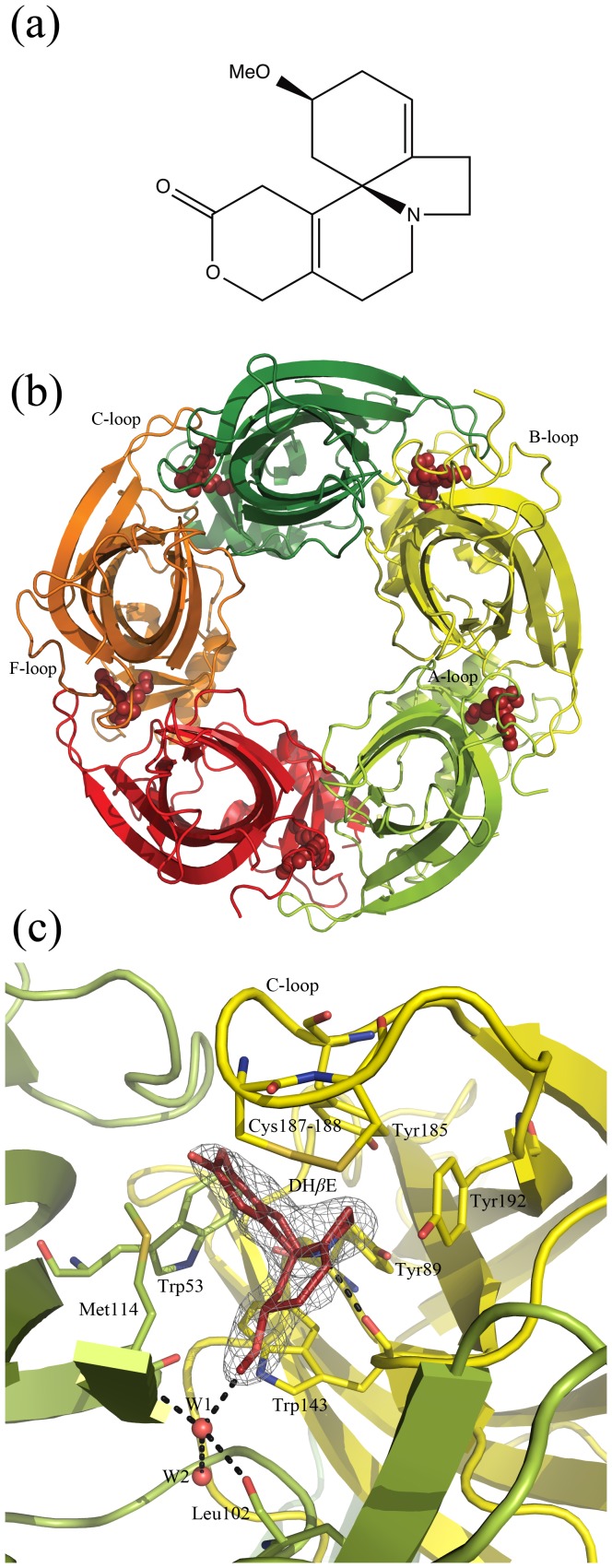
The structure of DH*β*E and *Ls*-AChBP complexed with DH*β*E. (*a*) Structure of DH*β*E. (*b*) Cartoon diagram showing homopentameric *Ls*-AChBP viewed along the five-fold symmetry axis. The five subunits are shown in different colors and DH*β*E in red spheres representation. (*c*) Ligand-binding pocket at the interface of two monomers formed by the highly conserved aromatic residues Tyr89, Trp143, Tyr185, and Tyr192 from the principal side of the interface (yellow) and Trp53 from the complementary side (limon). DH*β*E is shown in red and an omit 2Fo-Fc map is shown at 1*σ*. Hydrogen bonds between DH*β*E and its surroundings are shown as stippled lines. A blow-up of DH*ß*E and the omit 2Fo-Fc map shown at 1*σ* is provided in Fig. S2.

The erythrina alkaloid dihydro-*β*-erythroidine (DH*β*E) (Fig. 1a) is a potent competitive antagonist at nAChRs that has been used extensively as a pharmacological tool compound to gain a better understanding of the involvement of these receptors in physiological processes. DH*β*E is a somewhat selective antagonist with preference for *α*4 containing receptors [13]–[16]. It inhibits *α*4*β*2 receptors with nanomolar affinity (*K_i_* = 98 *n*M) [17] whereas affinities at *α*7 and *α*3*β*4 nAChRs lie in the micromolar range (*K_i_* = 11 and 32 µM, respectively) [17], [18].

To gain further insight into the inhibitory mechanism and binding mode of DH*β*E, we have determined the crystal structure of *Ls*-AChBP bound to DH*β*E. The structure reveals features that are unique to this antagonist.

## Results and Discussion

### DH*β*E binds at *Ls*-AChBP with an affinity comparable to that at *α*4*β*2 nAChRs

The binding affinity (*K_i_*) of DH*β*E at *Ls*-AChBP was determined to 52±5 *n*M by replacement of [^3^H]-epibatidine binding using a recently reported assay where *Ls*-AChBP for reasons of compatibility with other medium throughput assays was fused to a 5-HT3A ion channel [12]. The affinity of DH*β*E at *Ls*-AChBP closely resembles that of *α*4*β*2 nAChRs, supporting previous observations that *Ls*-AChBP can be used as a structural surrogate for *α*4*β*2 receptors [12] to study how DH*β*E interacts with the receptor.

### The structure of *Ls*-AChBP complexed with DH*β*E

The structure of *Ls*-AChBP was determined at 2.5 Å resolution (Table 1). The crystal belongs to space group *P*2_1_2_1_2_1_ with a DH*β*E molecule bound at the interface of all ten monomers in the asymmetric unit of the crystal. The DH*β*E-bound structure reported here shows the same homopentameric assembly as previously determined AChBP structures (Fig. 1b) [7]–[12]. Each monomer consists of an *N*-terminal *α*-helix, two short *α*3_10_ helices and a 10-stranded *β*-sandwich core. The F-loop portion of the molecule (residues 154–160 in subunits B and E and 155–160 in subunits C, G, H, I, and J) is not completely modeled due to lack of clear electron density, signifying a greater flexibility of these parts of the protein.

**Table 1:** Data collection and refinement statistics of the DH*β*E-bound *Ls*-AChBP structure.

Space group	*P*2_1_2_1_2_1_
Unit cell:	
*a*, Å	119.25
*b*, Å	121.31
*c*, Å	152.07
*α = ß = γ*, °	90.00
Resolution range, Å	19.61-2.51 (2.64-2.51)^a^
Completeness, %	98.5 (92.7)
Overall number of reflections	304,953
Number of unique reflections	74,987
Redundancy	4.1 (4.0)
R_merge_, %	7.4 (41.0)
*I/σI*	15.9 (2.0)
Solvent, %	43.7
Number of atoms	17,183
Number of DH*β*E molecules	10
Number of DH*β*E atoms	20
Number of water molecules	687
*R*_work_, %	20.0
*R*_free_, %	25.0
Ramachandran plot, residues in most favored regions, %	91.7
Rmsd of bonds lengths, Å	0.013
Rmsd of bonds angles, °	1.4
Average B-factor of protein main chains, Å^2^	35
Average B-factor of protein side chains, Å^2^	39
Average B-factor of water molecules, Å^2^	36
Average B-factor of DH*β*E molecules, Å^2^	30
Wilson B-factor, Å^2^	45

a Numbers in parentheses represent the last resolution shell values.

**Figure 2:**
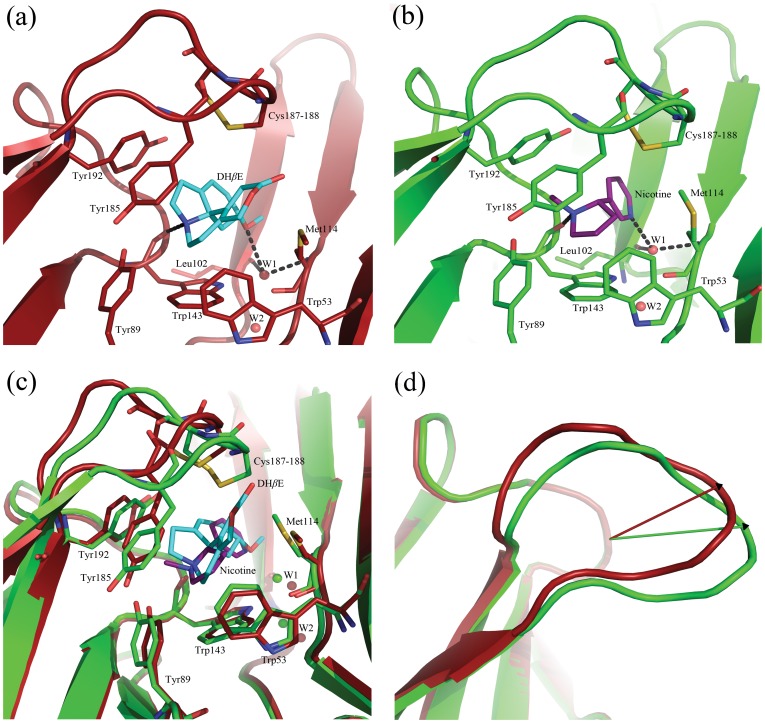
Comparison of DH*β*E-bound and nicotine-bound structures of *Ls*-AChBP. (*a,b*) Comparison of the ligand-binding site of the DH*β*E-bound structure (*a*, red) with the nicotine-bound structure (*b*, green). DH*β*E and nicotine are colored in cyan and purple, respectively. Hydrogen bonds between ligand and its surroundings are shown as stippled lines. The location of the residues is identical except for the residues from the C-loop (residues 185–192). Also, the conformation of the Met114 side chain from the complementary side is different between the two structures. (*c*) Conformational change of the C-loop due to DH*β*E binding to *Ls*-AChBP. The DH*β*E-bound structure (red) has been superimposed onto the nicotine-bound *Ls*-AChBP structure (green). (*d*) The projection vectors belonging to the nicotine-bound and DH*β*E-bound *Ls*-AChBP structures are shown in green and red, respectively. The angle between the two projection vectors is 21.4°. Angles between projection vectors of *Ls*-AChBP co-crystallized with nAChR agonists are listed in Table 2 for comparison. For further details, see Fig. S3.

The electron density map clearly demonstrate the existence of a single binding orientation for each of the ten DH*β*E molecules (Fig. 1c). DH*β*E binds underneath a closed C-loop at a position corresponding to that of nicotine in the *Ls*-AChBP crystal structure (PDB ID: 1uw6 [19]), Figs. 2a,b. The binding pocket is formed by the highly conserved aromatic residues Tyr89, Trp143, Tyr185, and Tyr192 from the principal side of the interface and Trp53 from the complementary side (Figs. 1c and 2a). This orientation is in agreement with a previous study where substitution of *β*2Trp82, *α*4Tyr126, *α*4Trp182, *α*4Tyr223, and *α*4Tyr230 in the *α*4*β*2 nAChR (corresponding to Trp53, Tyr89, Trp143, Tyr192, and Tyr195 in AChBP) for alanine were shown to decrease sensitivity to inhibition by DH*β*E [17].

Superposition of the nicotine-bound *Ls*-AChBP structure onto the DH*β*E-bound structure (C*^α^* atoms) gives a low RMSD value of 0.14 Å, indicating high structural similarity between the two structures. The main structural difference is the orientation of the C-loop (residues 185–192) (Fig. 2) capping the binding site. Also, the orientation of Met114 on the complementary side of the ligand-binding site differs between the two structures.

### DH*β*E shows a similar hydrogen-bonding network to agonists

The protonated tertiary nitrogen of DH*β*E lies within hydrogen-bonding distance of the backbone carbonyl of Trp143 (2.7 Å, chain D) and is also in close contact with the hydroxyl group of Tyr89 from the B-loop (3.5 Å, chain D) (Fig. 2a). Hydrogen bonds to these two residues on the principal side of the subunit interface have been observed for other agonists bound to AChBP [12], [19]. On the complementary side, DH*β*E interacts with the protein main chain *via* a water-mediated hydrogen bond (Fig. 2a). The oxygen of the methoxy group of DH*β*E accepts a hydrogen bond from a water molecule, which is tightly coordinated to the backbone carbonyl oxygen of Leu102 and nitrogen of Met114. Similar water-mediated contacts have been previously reported in agonist-bound structures (Fig. 2b) [7]–[9], [12], [19], [20]. In this way, the antagonist DH*β*E bridges the principal and complementary side of the interface in a way comparable to that of agonists.

### DH*β*E binds to *Ls*-AChBP under a shifted C-loop conformation

The conformation of the C-loop is very similar in all ten subunits (Fig. S1). In a previous study, the distance between the carbonyl oxygen atom of the conserved Trp residue in the A-loop (Trp143 in *Ls*-AChBP) and the *γ*-sulfur atom of the first Cys residue involved in disulfide bridge formation in the C-loop (Cys187 in *Ls*-AChBP) was used to quantify C-loop closure. This measurement was then correlated to the pharmacological profile of compounds co-crystallized with AChBP, suggesting a preference for antagonists to bind under open (extended) C-loops and agonists under closed (contracted) C-loops while partial agonists would bind under loops with intermediate closure [21]. Applying this metric to our DH*β*E-bound structure would classify the ligand as an agonist (Table 2), suggesting that it is insufficient to assess pharmacological fingerprints based on AChBP C-loop closure alone.

**Table 2:** Quantification of the C-loop conformational change.

*Ls*-AChBP complexed with	Trp143(O) - Cys187(S) distance (Å)^a^	Angle between projection vectors (°)^b^
Agonist: nicotine^c^	7.3	-
Agonist: carbamylcholine^d^	7.3	−5.0
Agonist: 1-(5-phenylpyridin-3- yl)-1,4-diazepane^e^	7.5	−4.2
Agonist: imidacloprid^f^	10.8	0.1
Agonist: 1-(5-ethoxypyridin-3-yl) 1,4- diazepane^g^	7.8	0.1
Agonist: 1-(6-bromopyridin-3-yl)-1,4-diazepane^h^	7.3	0.2
Agonist: 1-(6-bromo-5-ethoxypyridin-3-yl)-1,4- diazepane^i^	7.5	2.2
Agonist: 1-(pyridin-3-yl)-1,4-diazepane^j^	7.5	4.7
Agonist: clothianidine^k^	7.3	7.7
Antagonist: DH*β*E^l^	7.5	21.4

a Quantification of C-loop closure by the method of Brams *et al*. [21].

b For explanation on projection vectors, see Materials and Methods.

PDB ID (chain A):

c 1uw6;

d 1uv6;

e 3u8l (chain B);

f 2zju;

g 3u8k;

h 3u8m;

i 3u8n;

j 3u8j;

k 2zjv;

l 4alx.

Comparison of DH*β*E-bound *Ls*-AChBP with previously determined structures of *Ls*-AChBP in complex with small molecule agonists shows a new conformational state of the C-loop, which is not reflected by the distance measurement discussed above. This conformational state has not been observed in previously reported AChBP structures, where a closed C-loop corresponds to an agonist-bound state and an open C-loop to an antagonist-bound state. The C-loop conformational change has been quantified by measuring the angle between the projection of a vector defined from the center of the C-loop to the C*^α^* atom of Cys187 in the DH*β*E-bound structure, and the corresponding projection vector in the nicotine-bound structure, which has been used as reference (Table 2, Fig. 2d). For further explanation on projection vectors, see Materials and Methods. The angle in the DH*β*E-bound structure is 21.4°, while this number lies within the range of −5°–7.7° for all other *Ls*-AChBP structures complexed with different agonists. These measurements reveal that the C-loop undergoes a conformational movement, which is perpendicular to the previously observed C-loop movements in AChBP structures and thus could indicate that DH*β*E inhibits nAChRs by a unique mechanism.

To investigate if this C-loop movement could be due to crystal packing effects, we undertook a detailed analysis of the DH*β*E-bound structure. Only C-loops of five subunits (chains B and D of one pentamer and chains G, I, and J of the other pentamer) out of ten subunits of the *Ls*-AChBP structure in complex with DH*β*E are in contact (closer than 3.5 Å) with symmetry-related molecules. Furthermore, different loop regions (residues 23–28, 67–72 or 160–167) of the symmetry-related molecules are involved in those contacts. Therefore, it is unlikely that the difference in C-loop conformation of the DH*β*E-bound structure compared to other *Ls*-AChBP structures is determined by crystal packing forces. To investigate C-loop flexibility, we compared the average C-loop B-factor to the average B-factor of all protein atoms. The average B-factor of all ten C-loops in the DH*β*E-bound structure is 47 Å^2^ compared to 37 Å^2^ for all protein atoms. Thus, the average B-factor is slightly increased at the C-loop relative to the overall average B-factor. However, other *Ls*-AChBP structures in complex with agonists (PDB IDs: 1uv6, 3u8l, 2zju, 3u8k, 3u8m, 3u8n, and 2zjv) show the same trend as for the DH*β*E-bound structure, except for the complexes with nicotine (PDB ID: 1uw6) and NS3531 (PDB ID: 3u8j). In these latter two structures, the C-loop has lower and equal values, respectively, compared to the overall average B-factor.

A similar hypothesis that DH*β*E inhibits nAChRs by a unique mechanism was previously raised by Bertrand *et al*. Based on electrophysiological data [22] it was shown that an L247T mutation in the *α*7 nAChR ion channel domain renders DH*β*E an agonist [22]. Since mutation of L247T also reduces desensitization, it was suggested that DH*β*E inhibits the activity of nAChRs by stabilizing the desensitized state rather than the non-activated state of the receptor. The unique conformation of the C-loop observed in the DH*β*E-bound structure of *Ls*-AChBP together with a hydrogen-bonding network similar to that seen for agonists supports a unique mode of antagonism for DH*β*E compared to prototypical antagonists and toxins.

## Conclusions

In this study, we have determined the crystal structure of *Ls*-AChBP in complex with DH*β*E, which is a potent competitive antagonist of nAChRs. The structure reveals three main features that are unique to this antagonist: (*i*) DH*β*E introduces a C-loop closure compared to that of agonists, (*ii*) the C-loop undergoes a conformational shift perpendicular to the previously observed C-loop movements, and (*iii*) the hydrogen-bonding network of DH*β*E is similar to that of agonists. Thus, DH*β*E seems to prevent receptor activation *via* a mechanism different from that of prototypical antagonists and toxins.

## Materials and Methods

### Protein purification and crystallization

Recombinant *Ls*-AChBP was expressed using the Bac-to-Bac baculovirus expression system in *Sf*9 insect cells and purified as described previously [12], [19]. The protein solution was incubated with 50 mM DH*β*E prior to crystallization. DH*β*E-bound crystals were obtained using the hanging drop vapor diffusion method at 20°C. Crystallization drops were made by mixing 1 µl of a 4.9 mg/ml protein:DH*β*E solution in 20 mM Tris Base (pH 8.0) and 20 mM NaCl with 1 µl of crystallization solution containing 0.1 M HEPES (pH 7.5), 25% v/v polyethylene glycol (PEG) 400, and 0.2 M MgCl_2_. Crystals grew within 3 weeks to a final length of 0.2 mm.

### Crystallographic data collection, refinement, and model building

The crystal was mounted in a cryo-loop and flash-cooled in liquid nitrogen after brief immersion in a cryo-protectant composed of mother liquor supplemented with 25% (v/v) glycerol. X-ray data were collected at 100 K on beamline I911-3 at the MAX-lab synchrotron, Lund, Sweden, using a marmosaic 225 detector at a wavelength of 0.997 Å. Data were processed and scaled using *XDS* [23] and *Scala* [24], respectively. Five percent of the data were set aside during the scaling process as test set for calculation of *R*_free_.

The structure was solved by the molecular replacement method using the program *Phaser* [25]. A pentamer of *Ls-*AChBP (in-house structure; to be published) was used as the search model. The refinements were performed with *Phenix* [26] using non-crystallographic symmetry (NCS) and rebuilt interactively using *Coot* [27]. Residues in the F-loop (154–164) were excluded from NCS restraints. The input structure of DH*β*E was generated using Maestro [28] and MacroModel [29]. Low energy conformations of DH*β*E with a protonated tertiary nitrogen were generated using the Monte Carlo molecular mechanics method with an energy cutoff set to 13 kJ/mol and used to generate geometry restraints after selection of the low energy conformer with the best visual fit to the electron density map. Water molecules were added during the refinement using *Phenix*. The starting *R*_work_ and *R*_free_ of the structure were 39.1% and 40%, respectively, which were improved to the final *R*_work_ and *R*_free_ of 20.1% and 24.2%, respectively. Data collection and refinement statistics are summarized in Table 1.

The quality of the final model was assessed by examination of the detailed stereochemistry using *Procheck* [30] and *Molprobity* [31]. The Ramachandran plot of the structure shows that 91.7% of the residues are in the most favored regions by the *Procheck* criteria, 8.1% in additionally allowed regions and 0.2% in the generously allowed regions.

### Structure analysis

The structure and ligand analyses were performed using *Coot* [27] and *PyMOL* [32]. The figures were generated using *PyMOL*.

The superposition of the DH*β*E-bound structure onto the nicotine-bound *Ls*-AChBP structure was performed on C*^α^* atoms employing the “super” command in *PyMOL*. The projection vector (shown in green, Fig. 2d) belonging to the nicotine-bound structure was defined as follows: (*i*) First, a reference plane was defined at the C-loop position (residues 185–192) in the nicotine-bound structure (PDB ID: 1uw6, chain A [19]). (*ii*) The vector defined between the center of the reference plane and the C*^α^* atom of Cys187 in the nicotine-bound structure was projected onto the reference plane to define the reference projection vector (shown in green, Fig. 2d). The projection vector belonging to the DH*β*E-bound structure (shown in red, Fig. 2d) was defined as follows: (*i*) The structure of DH*β*E-bound *Ls*-AChBP (chain A) was superimposed onto the nicotine-bound reference structure, based on residues located in the central *β-*sheets and C*^α^* atoms. (*ii*) The vector defined between the center of the reference plane in the nicotine-bound structure and the C*^α^* atom of Cys187 in the DH*β*E-bound structure was projected onto the reference plane, defining the projection vector (shown in red, Fig. 2d).

### Accsession Numbers

Coordinates and structure factors have been deposited in the Protein Data Bank with accession number 4alx.

## Supporting Information

Figure S1 The conformation of the C-loop is very similar in all ten subunits. The subunits of the DH*β*E-bound structure have been superimposed (shown in different colors).
(TIF)

Figure S2 An omit 2Fo-Fc map for DH*β*E shown at 1*σ* with DH*β*E modeled in. Three different views of the electron density are shown.
(TIF)

Figure S3 Vector representation showing the conformational change of the C-loop due to DH*β*E binding to *Ls*-AChBP. The DH*β*E-bound structure (red) has been superimposed onto the nicotine-bound *Ls*-AChBP structure (green). **Ob** is the distance between the center of the C-loop and C*^α^* atom of Cys187 in DH*β*E-bound *Ls*-AChBP structure, and **Oa** is the corresponding projection vector. **Oc** is the distance between the center of the C-loop and C*^α^* atom of Cys187 in nicotine-bound *Ls*-AChBP structure, and **Od** is the corresponding projection vector.
(TIF)
