# Supplementary figures and images for: Crystal Structure of Lymnaea stagnalis AChBP Complexed with the Potent nAChR Antagonist DHβE Suggests a Unique Mode of Antagonism
Source: PLoS One. 2012 Aug 22;7(8):e40757. doi: 10.1371/journal.pone.0040757 (PMC3425559; doi:10.1371/journal.pone.0040757)

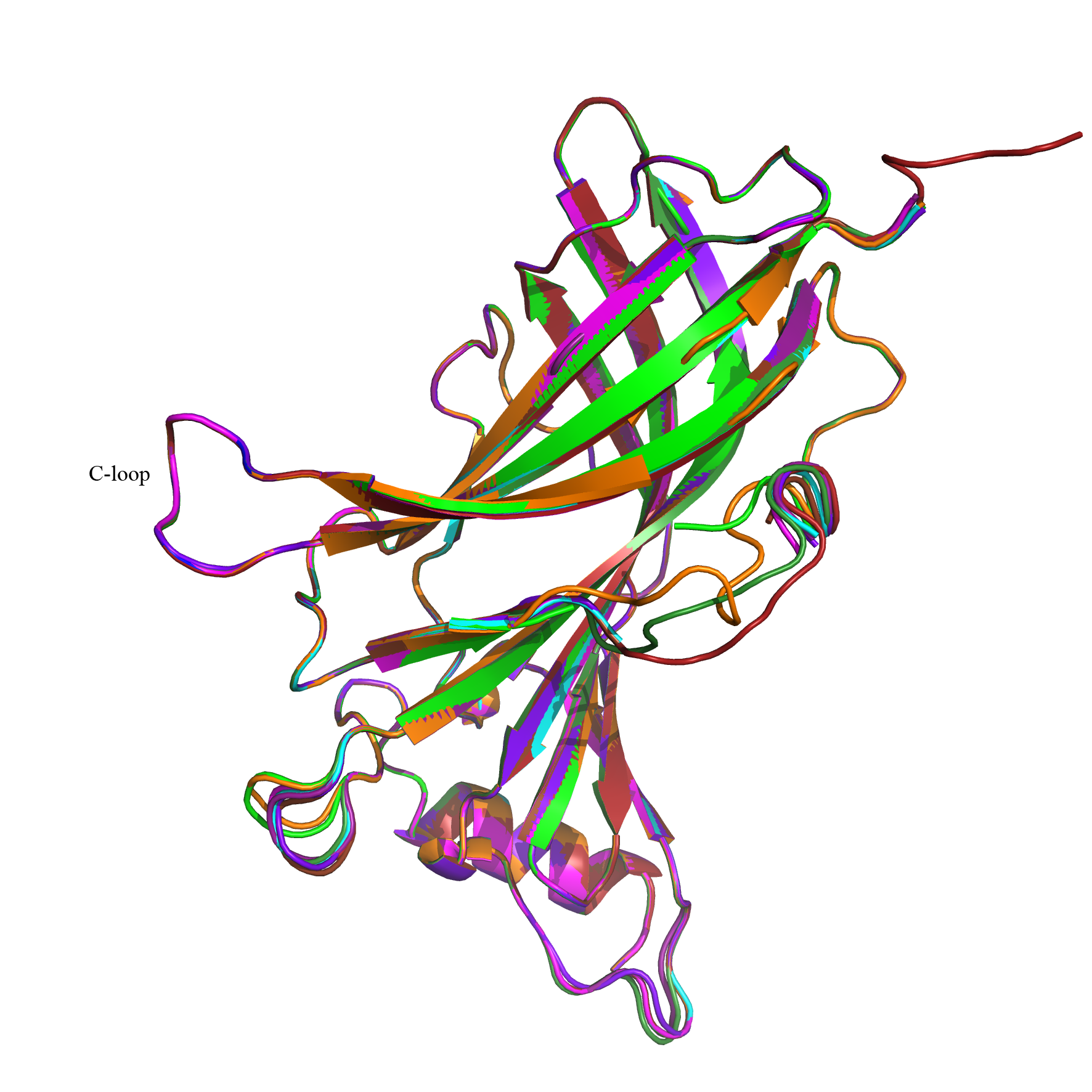

Supplement: Figure S1 — The conformation of the C-loop is very similar in all ten subunits. The subunits of the DHβE-bound structure have been superimposed (shown in different colors). (TIF) [file pone.0040757.s001.tif]

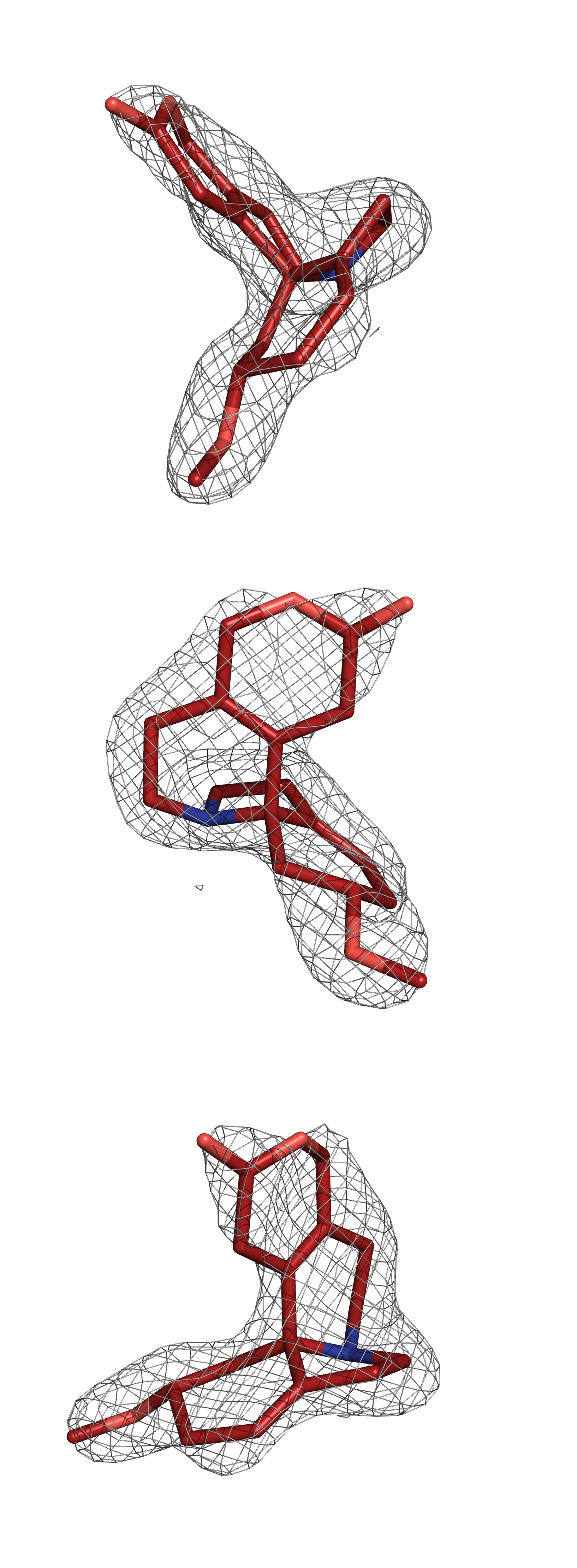

Supplement: Figure S2 — An omit 2Fo-Fc map for DHβE shown at 1σ with DHβE modeled in. Three different views of the electron density are shown. (TIF) [file pone.0040757.s002.tif]

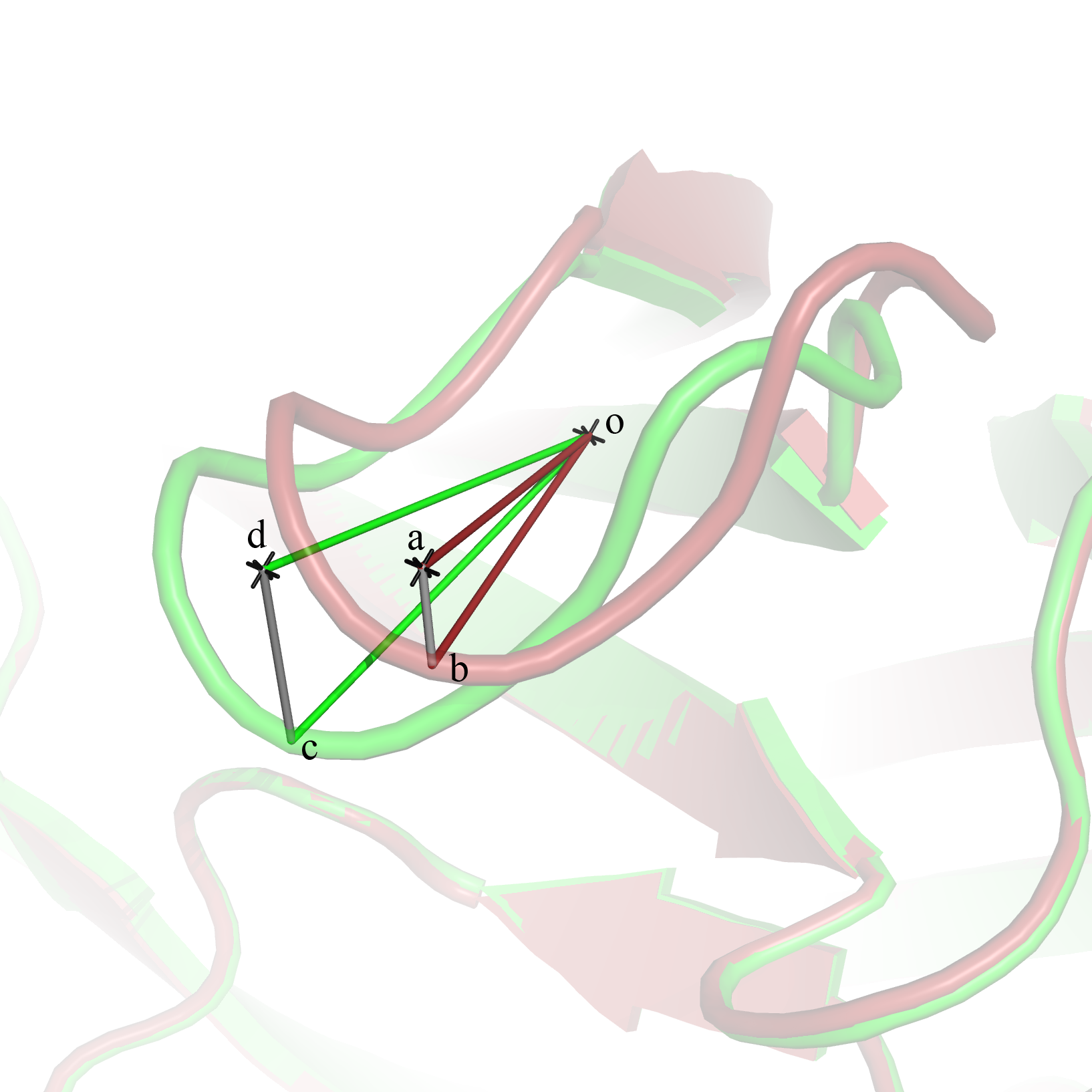

Supplement: Figure S3 — Vector representation showing the conformational change of the C-loop due to DHβE binding to Ls-AChBP. The DHβE-bound structure (red) has been superimposed onto the nicotine-bound Ls-AChBP structure (green). Ob is the distance between the center of the C-loop and Cα atom of Cys187 in DHβE-bound Ls-AChBP structure, and Oa is the corresponding projection vector. Oc is the distance between the center of the C-loop and Cα atom of Cys187 in nicotine-bound Ls-AChBP structure, and Od is the corresponding projection vector. (TIF) [file pone.0040757.s003.tif]
